# Variations in cognition by human capital characteristics: a cross-sectional analysis of Brazilian older adults

**DOI:** 10.3389/fpubh.2023.1257961

**Published:** 2023-10-24

**Authors:** Paolo Miguel Manalang Vicerra

**Affiliations:** Asian Demographic Research Institute, Shanghai University, Shanghai, China

**Keywords:** health disparity, socioeconomic characteristics, population heterogeneity, cognitive performance, social determinants of health

## Abstract

**Introduction:**

The productivity of individuals is assumed to decline upon reaching old age when cognitive ability is considered. This assumption is false, especially if the human development characteristics of people are analyzed, which highlights the need to recognize the heterogeneity among subpopulations.

**Methods:**

Using Wave two of the Brazilian Longitudinal Study on Aging, conducted from 2019 to 2021, this study explored the onset and speed of cognitive aging among older individuals aged at least 60 in Brazil, with reference to their education and income levels.

**Results:**

It was observed that although higher human capital characteristics yielded results toward later cognitive decline, women benefited more from having higher educational attainment levels. Such a pattern was similar among men and increased income levels. Disparities in cognitive performance, whether from education or income, were greatest at age 60, and this advantage diminished as age progressed.

**Conclusion:**

Viewing the older population as homogeneous in terms of health function is restrictive. It should be recognized that variations in social status affect individuals’ health status into old age and therefore their respective potential for productivity should be maximized.

## 1. Introduction

Improvements in social conditions, better living environments, and advances in medical science have resulted in increased life expectancies across countries (1). In conjunction with this, the phenomenon of population aging has been observed in many societies. Brazil is among such cases, where the median age of the population has increased from 16.9 years in 1960 to 32.4 years in 2020 (2). It is estimated to reach 46 by 2060. In terms of proportional distribution in 1960, a little over 4% of Brazil’s population were aged 60 and over, while those in the younger age groups of 0 to 14 comprised 38% of the total population. By 2020 and 2060, the older population is estimated to make up about 14 and 33%, respectively, of the total population. During those same periods, the younger age groups will account for almost 21 and 14%, respectively, of the total population.

The onset of ill health is associated with age progression, including declining cognitive performance, notwithstanding dementia (3). There is a biological underpinning in this process as the brain and its functions degenerate in advanced ages (4). Additionally, social disparities can be involved in outcomes pertaining to cognition (5). In 2015, it was estimated that about 5% of the global older population had dementia (3). A higher prevalence of dementia has also been estimated to occur in low- and middle-income countries (LMICs), which constitute around 60% of the global prevalence. In the case of Brazil, dementia is said to be underdiagnosed, with an estimated number of about a million affected individuals (6). Studies at the community level also indicate widely varying prevalence rates between 5 and 19% of the respective communities’ older populations (7).

The shift in age structures of societies towards aging has been linked to public health challenges, as there may be issues related to financing healthcare and ensuring a good quality of well-being (1). There are studies that have noted the prospect that such a change in population structure may lead to negative population growth and labor scarcity, with China serving as an example (8). However, recent studies challenge this point of view by delving into the fact that the ‘older population’ is highly heterogeneous, with individuals having different socioeconomic backgrounds. Therefore, their health and capacity to contribute to society in an economically gainful manner can continue despite old age (9–11). Cognition plays a crucial role in the productivity of the older population, serving as an apt indicator for further social engagement and adaptability in learning and navigating work environments.

This paper employs the previously presented notion of heterogeneity. The entrance to old age should not be defined by chronological age alone, as it does not signify the level of health functioning of individuals. With the increase in life expectancies across societies, the health status and functioning of people today differ significantly from those decades ago. For example, hypothetically, a 60-year-old man today may have the health functioning capacity of a 50-year-old male in the 1980s. This fundamental concept is put forth through the characteristics approach, which is multidimensional and determines the functional capacity of individuals based on chronological age and social characteristics (12). As a hypothetical example, a female aged 65 with no completed schooling may exhibit a cognitive performance level of X, whereas a woman with completed secondary school attainment may exhibit the same level of cognitive functioning X at 70 years old. This implies that individuals with a higher socioeconomic background may experience a later onset of cognitive aging. The characteristics approach has been applied to various health dimensions, including walking speed, handgrip strength, and cognitive status (13–15).

The present study focuses on the differences in cognitive aging among subpopulations in Brazil. As the rate of population aging in the country is increasing, several general social and economic issues have been raised, often overlooking the aspect of cognitive impairment in the discussion (6, 7). As individuals experience ageing differently, this study can provide guidance on which individuals in the population may maintain cognitive productivity. Having an aging population may not only bring social and economic challenges but also offer opportunities when taking a positive perspective and appropriate consideration.

## 2. Materials and methods

### 2.1. Data

This study utilized the second wave of the Brazilian Longitudinal Study on Aging (ELSI-Brazil), which includes a total sample of 9,941 participants from whom information was collected between 2019 and 2021. ELSI-Brazil is an ongoing nationally representative cohort study that focuses on individuals aged at least 50 years, with the baseline data collected in 2015–2016. The collected data includes socio-demographic characteristics, self-reported health measures, and anthropometric data. ELSI-Brazil implemented a complex sampling design, and additional details regarding the survey methodology can be found elsewhere (16). The ELSI-Brazil was approved by the Ethics Committee of the Oswaldo Cruz Foundation - Minas Gerais and the process is registered on Plataforma Brasil (CAAE: 34649814.3.0000.5091). Participants signed separate informed consent forms for each of the research procedures and authorized access to corresponding secondary databases.

### 2.2. Measures

#### 2.2.1. Cognition

A global score was generated from two measures of cognition. The first measure involved immediate and delayed recall. Respondents were presented with a list of 10 words and asked to recite them back to the survey enumerator immediately. After answering other unrelated questions, the respondents were asked again to recite the same 10 words. The second measure of cognition was fluency, specifically involving naming as many animals as possible within a minute. The total number of correct answers was combined to represent the global cognition score, following a similar approach as other studies (17, 18).

#### 2.2.2. Human capital factors

The characteristics focused on in the analyses were socioeconomic factors: education and income. Education was determined based on the survey item that asked about the highest level of education attained by individuals. The categories used were as follows: (a) no education, (b) primary level, (c) secondary level, and (d) postsecondary level. Regardless of completion status, respondents were included in their respective classification. For the characteristics approach analysis, the reference category for comparison was the category of no education.

Income was based on the total household income from various sources reported by the respective older respondents. The total income values were then classified into quartiles. The reference category for income was the lowest quartile.

#### 2.2.3. Other covariates

In previous applications of the characteristics approach, additional health variables that were correlated with the investigated health outcome were included (11, 13). The bases for the selection of the said health covariates are their observed association with the outcome at hand and also that they are contextualized in the society being investigated. With that said, three medically-diagnosed health status factors were included in the analyses: hypertension, arthritis, diabetes, a cerebral vascular accident or stroke, and chronic obstructive pulmonary disease (COPD). The prevalence of the said diseases has been indicated to be associated with cognitive performance decline among Brazilians over the years (19–21). An advantage of utilizing the characteristics approach as an analytical method is its concise statistical modelling, but the prevalence rates of the previously mentioned diseases were utilized in the present analysis to account for the health status of the analytic sample. The goal was to produce better estimates with regard to the adjusted rates of human capital factors similar to what was done in previous studies involving the characteristics approach (11, 13–15).

### 2.3. Analysis

The characteristics approach is initiated by creating a schedule of health functions using multiple linear regression models (13, 14). The outcome of these analytical models is cognitive performance, which depends on individual ages and human capital development characteristics. The distribution of cognitive performance characteristics is non-normal, but despite this, the use of the stated regression model analyses is acceptable as violations of the normality assumption occur due to small sample sizes (22). This method of analysis, which yields outcomes with a non-normal distribution, has been utilized in other studies (14, 15). Distinct analytic models were employed for education levels and income quartiles. The model specification for education is:


$$\begin{array}{l} C{\left(k\right)}_{i}=\beta_{0}+\beta_{1}{\text{age}}^{2}+\beta_{2}\text{Primary}+\beta_{3}\text{Secondary}\\ \;+\beta_{4}\text{Postsecondary}+\beta_{5}\text{Hypertension}+\beta_{6}\text{Arthritis}\\ \;+\beta_{7}\text{Diabetes}+\beta_{8}\text{Stroke}+\beta_{9}\text{COPD}+\mu_{i} \end{array}$$

And the model specification for income quartile analysis was:


$$\begin{array}{l} C{\left(k\right)}_{i}=\beta_{0}+\beta_{1}{\text{age}}^{2}+\beta_{2}\text{Secondquartile}+\beta_{3}\text{Thirdquartile}\\ \;+\beta_{4}\text{High}+\beta_{5}\text{Hypertension}+\beta_{6}\text{Arthritis}\\ \;+\beta_{7}\text{Diabetes}+\beta_{8}\text{Stroke}+\beta_{9}\text{COPD}+\mu_{i} \end{array}$$

where *C*(*k*) stands for the health status *k* referring to cognition level of person *i*. Then ε_i_ is the random error with zero mean and the variance σ^2^.

The next step in the characteristics approach is computing α-ages, or alpha ages. These are characteristic-based ages, where individuals showing the same α-age have the same characteristic (13). A standard schedule is established, which serves as the comparative reference for characteristics based on chronological age. In the present analysis, the reference category for education level was individuals with no schooling. As for the income quartile, the reference category was the low-income category. The following equations were for the α-ages of education subgroups:


$$\alpha_{k,\text{Primary}}=\sqrt{\text{ag}e_{\text{Noeduc}}^{2}-\;\left({\hat{\beta }}_{2}/{\hat{\beta }}_{1}\right)\;}$$

$$\alpha_{k,\text{Secondary}}=\sqrt{\text{ag}e_{\text{Noeduc}}^{2}-\;\left({\hat{\beta }}_{3}/{\hat{\beta }}_{1}\right)\;}$$

$$\alpha_{k,\text{Postsecondary}}=\sqrt{\text{ag}e_{\text{Noeduc}}^{2}-\;\left({\hat{\beta }}_{4}/{\hat{\beta }}_{1}\right)\;}$$

For the α-ages of the subpopulations based on income:


$$\alpha_{k,\text{Secondquartile}}=\sqrt{\text{ag}e_{\text{Low}}^{2}-\;\left({\hat{\beta }}_{2}/{\hat{\beta }}_{1}\right)\;}$$

$$\alpha_{k,\text{Thirdquartile}}=\sqrt{\text{ag}e_{Low}^{2}-\;\left({\hat{\beta }}_{3}/{\hat{\beta }}_{1}\right)\;}$$

$$\alpha_{k,\text{High}}=\sqrt{\text{ag}e_{\text{Low}}^{2}-\;\left({\hat{\beta }}_{4}/{\hat{\beta }}_{1}\right)\;}$$

## 3. Results

The sample distribution is presented in Table 1. The mean age and cognition levels of men and women in the sample were similar. There were more men at the primary level of education, while there were more women at the other levels. In terms of income quartile, more women were in the lower categories, while men were in the higher levels.

**Table 1:** Descriptive statistics of analytic sample by gender.

	Total (*n* = 4,099)	Men (*n* = 1,646)	Women (*n* = 2,453)
Mean age	69.4	69.5	69.4
Mean cognition	17.4	17.6	17.3
Age group (%)			
60–69	58.8	58.3	59.2
70–79	30.3	29.8	30.8
80 and over	10.9	11.9	10.0
Education level (%)			
No schooling	15.9	14.9	16.7
Primary	51.6	53.3	50.2
Secondary	25.6	25.2	26.0
Postsecondary	6.9	6.6	7.1
Income quartile (%)			
Low	21.3	18.7	23.4
Second	30.6	28.8	32.2
Third	20.3	21.8	19.1
High	27.8	30.8	25.3
Hypertension prevalence (%)	14.6	16.3	13.2
Arthritis prevalence (%)	21.7	14.1	28.1
Diabetes prevalence (%)	20.1	18.3	21.7
Stroke prevalence (%)	4.1	4.7	3.6
Chronic obstructive pulmonary disease prevalence (%)	3.7	3.9	3.5

Source: Brazilian longitudinal study on aging (ELSI-Brazil), 2019–2021.

Table 2 presents the differences in average global cognition scores between men and women. It was observed that average cognition scores decrease with older ages. Men with no completed education had an average score of about 15, while women had an average score of 14. For those at the postsecondary level, the average scores for both sexes were almost 23. Similarly, cognition scores increased as human capital development levels increased.

**Table 2:** Comparison of mean global cognition scores between men and women.

	Men	Women	*p*-value^+^
Age group			
60–69	18.29	18.08	0.531
70–79	17.10	16.58	0.257
80 and over	15.70	14.75	0.174
Education attainment			
No schooling	14.72	13.85	0.073
Primary	16.55	16.15	0.236
Secondary	20.28	20.16	0.813
Postsecondary	22.83	22.83	0.997
Income quartile			
Low	16.26	15.83	0.393
Second	17.15	16.69	0.264
Third	18.28	17.16	0.081
High	18.44	19.48	0.039

+ Based on *t*-test.

**Figure 1:**
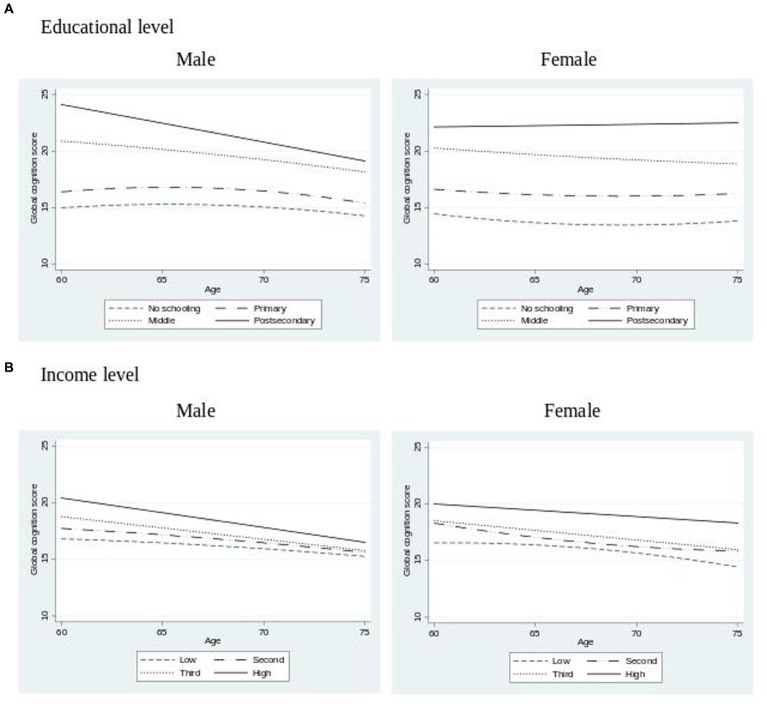
Mean cognition score by human capital development factors and gender.

To further explore differences in the trajectories of cognitive performance based on socioeconomic characteristics, the age progression of the health outcome is depicted in Figure 1. There is a significant disparity in average scores based on education levels. Regarding income categories, there are also significant disparities between categories, particularly at age 60. Among men, the differences tend to diminish with older ages. Women in the lower three categories of income showed smaller differences in scores, while the high-income quartile remained notably elevated throughout the age progression.

The results of the characteristics approach analyses by education level are presented in Table 3. Men at the primary level of education have a greater than 1-year advantage over those with no schooling. Higher gains were observed as education levels increased. For example, the level of cognitive performance of a 60-year-old male with no schooling would be attained at age 66 for those at the secondary level and 69 years old for those at the postsecondary level. A similar pattern was observed among women. Another similarity between men and women is that the gains decrease as age progresses. For instance, women with no schooling and those at the postsecondary level had alpha-ages of 60 and 72 years, respectively, resulting in a 12-year difference. At age 80, women with no schooling and women at the postsecondary level may have similar performances at age 89, reducing the difference to 9 years.

**Table 3:** Alpha-age schedule by educational attainment level of men and women.

	Male			Female		
Age with no schooling	Primary	Secondary	Postsecondary	Primary	Secondary	Postsecondary
60	61.90	66.64	69.09	63.15	68.32	72.11
65	66.76	71.16	73.46	67.91	72.75	76.31
70	71.64	75.76	77.93	72.71	77.24	80.63
75	76.51	80.41	82.45	77.53	81.80	85.00
80	81.42	85.098	87.03	82.39	86.40	89.44

The patterns and trends among men and women for α-ages based on income levels were similar to those based on education levels (Table 4). A notably consistent pattern between income and education levels was that women had higher gains than men for the highest categories. Specifically, women in the high-income category, as well as those at the postsecondary level, had approximately 20% higher gains in α-ages than men.

**Table 4:** Alpha-age schedule by income level and gender.

	Male			Female		
Age of low income level	Second	Third	High	Second	Third	High
60	62.93	67.19	68.96	62.87	65.95	71.48
65	67.73	71.67	73.35	67.65	70.53	75.73
70	72.54	76.24	77.82	72.47	75.16	80.07
75	77.36	80.87	82.35	77.31	79.84	84.46
80	82.22	85.51	86.93	82.16	84.55	88.93

## 4. Discussion

This study observed the cognitive performance of older Brazilian men and women and identified socioeconomic disparities. Higher levels of education and income were associated with a later onset of cognitive decline, benefiting cognition across the old age spectrum. However, a declining trend was observed upon reaching later ages for both males and females.

In general, socioeconomic factors have shown to have a positive impact on health, particularly in terms of cognition (23, 24). Education has been suggested to enhance cognitive reserves (25) and offer lifelong opportunities that can delay cognitive ageing (24). Greater economic resources are also associated with better healthcare access and social engagement, which can benefit cognitive health (26, 27). The range of activities that people can do when they have more resources, especially being engaged in community activities.

Similar patterns of better health status related to higher human capital characteristics have been observed in Brazil (28, 29). Illiteracy rates have been declining, but disparities persist among certain subpopulations, including older individuals (30). It has been reported that even within the older population, further disparities are noted. When viewed at the regional level, adults aged 60 and over had illiteracy rates of about 33% in the Northeast, while it is nearly 9% in the Southeast (30). Regarding income, social security schemes have been implemented to promote health and well-being (29). As a result of these programs, about 86% of older individuals in Brazil receive support through social pension schemes. As observed in the present study, the onset of cognitive decline among those with no schooling and low income was much disadvantaged.

Significant differences in the benefits toward cognitive performance were found between genders. Men and women exhibited different degrees of advantage depending on socioeconomic status. Across higher levels of education, women had a greater advantage. These results resemble findings from other LMICs (11, 15). In the context of the current study, women in Brazil have been found to experience inequality in terms of education, labor participation, and income (28, 31). Due to lower returns on income for women, they may compensate through other means (32). This may explain why the higher benefits in the onset of cognitive performance decline are consistently higher for women across all education levels.

There are limitations to this study. The use of cross-sectional data within the panel survey of ELSI-Brazil aligns with selected literature that utilizes the characteristics approach thus far (11). Given the nature of the data used, no causation can be established in the analysis. The results pertaining to the prevalence of cognitive performance also do not indicate a medical diagnosis of dementia. The survey items used are based on established questions for estimating cognitive performance levels. Lastly, the present study focused on the functional differences in the ageing process of the population based on human capital factors. Other studies have explored biomarker-based biological ages (33, 34) but such studies were complex and have extemporized assumptions (13). Furthermore, the present study focused on the general population and estimated the respective trajectories of cognitive performance by human capital development characteristics. Prospective studies may be able to estimate similar trajectories with the inclusion of their health status. It may be utilized as a stratifying characteristic of the analytic sample apart from health status factors as covariates in the statistical modelling analysis in the initial procedure of the characteristics approach.

## 5. Conclusion

By employing the characteristics approach as a novel analytic method, this study provides a better understanding of the heterogeneity among the older population. The onset and progression of cognitive decline were observed to be associated with human capital development factors. Both education and income levels are preventable risk factors for cognitive impairment. For the current older population, it may be necessary to monitor those with no schooling as they may be at a higher risk of cognitive decline. Healthcare and services should be better aligned with the needs of individuals based on their social and economic backgrounds. Looking toward the long term, measures should be strengthened to improve educational attainment levels for the current younger population. This would lead to increased prospective earnings and better cognitive health outcomes in old age.

## Data availability statement

Publicly available datasets were analyzed in this study. The data sets presented in this study can be found in online repositories. The names of the repository/repositories and accession number(s) can be found at https://elsi.cpqrr.fiocruz.br/data-access/.

## Author contributions

The author confirms being the sole contributor of this work and has approved it for publication.
